# Curcumin Analogue C1 Promotes Hex and Gal Recruitment to the Plasma Membrane via mTORC1-Independent TFEB Activation

**DOI:** 10.3390/ijms20061363

**Published:** 2019-03-18

**Authors:** Alessandro Magini, Alice Polchi, Danila Di Meo, Sandra Buratta, Elisabetta Chiaradia, Raimondo Germani, Carla Emiliani, Brunella Tancini

**Affiliations:** 1 Department of Chemistry, Biology and Biotechnology, University of Perugia, Via del Giochetto, 06122 Perugia, Italy; alice.polchi@gmail.com (A.P.); danila.dimeo@gmail.com (D.D.M.); sandra.buratta@unipg.it (S.B.); raimondo.germani@unipg.it (R.G.); carla.emiliani@unipg.it (C.E.); 2 Institute for Molecular Cell Biology, University of Münster, Schlossplatz 5, 48149 Münster, Germany; 3 Cells-in-Motion Cluster of Excellence, University of Münster, D-48149 Münster, Germany; 4 Department of Veterinary Medicine, University of Perugia, Via S. Costanzo 4, 06126 Perugia, Italy; elisabetta.chiaradia@unipg.it

**Keywords:** TFEB, curcumin, curcumin analogue C1, lysosomal glycohydrolases, plasma membrane-associated glycohydrolases

## Abstract

The monocarbonyl analogue of curcumin (1E,4E)-1,5-Bis(2-methoxyphenyl)penta-1,4-dien-3-one (C1) has been used as a specific activator of the master gene transcription factor EB (TFEB) to correlate the activation of this nuclear factor with the increased activity of lysosomal glycohydrolases and their recruitment to the cell surface. The presence of active lysosomal glycohydrolases associated with the lipid microdomains has been extensively demonstrated, and their role in glycosphingolipid (GSL) remodeling in both physiological and pathological conditions, such as neurodegenerative disorders, has been suggested. Here, we demonstrate that Jurkat cell stimulation elicits TFEB nuclear translocation and an increase of both the expression of hexosaminidase subunit beta (*HEXB*), hexosaminidase subunit alpha (*HEXA*), and galactosidase beta 1 (*GLB1*) genes, and the recruitment of β-hexosaminidase (Hex, EC 3.2.1.52) and β-galactosidase (Gal, EC 3.2.1.23) on lipid microdomains. Treatment of Jurkat cells with the curcumin analogue C1 also resulted in an increase of both lysosomal glycohydrolase activity and their targeting to the cell surface. Similar effects of C1 on lysosomal glycohydrolase expression and their recruitment to lipid microdomains was observed by treating the SH-SY5Y neuroblastoma cell line; the effects of C1 treatment were abolished by TFEB silencing. Together, these results clearly demonstrate the existence of a direct link between TFEB nuclear translocation and the transport of Hex and Gal from lysosomes to the plasma membrane.

## 1. Introduction

Transcription factor EB (TFEB) is a master regulator of many cellular processes, such as lysosomal functions, autophagy, and membrane repair [1,2,3,4]. Moreover, the involvement of TFEB in cell migration is clearly emerging [5,6,7].

TFEB recognizes and binds to a regulatory sequence, the coordinated lysosomal expression and regulation (CLEAR) motif, which is present in the promoter region of several lysosomal genes [8]. TFEB modulates and coordinates the main lysosome-dependent degradative pathways to promote intracellular clearance. TFEB activity depends on its phosphorylation status, which is mainly regulated by the mechanistic target of rapamycin complex 1 (mTORC1) and calcineurin, a Ca^2+^-dependent phosphatase [9,10]. Numerous studies support the idea that impaired TFEB function may contribute to the pathogenesis of most degenerative diseases characterized by aberrant intracellular accumulation of macromolecules [11,12,13]. Conversely, genetic or pharmacological activation of TFEB proved to be beneficial in a variety of neurodegenerative and lysosomal storage diseases (LSDs) [14,15,16].

Recently, our group demonstrated that TFEB activation is associated with the recruitment of mature acidic glycohydrolases to the plasma membrane by lysosomal exocytosis induction [17]. Currently, many reports indicate the presence of glycosyltransferases and glycohydrolases on the cell surface, and it has been suggested that these enzymes may play an important role in the in situ remodeling of glycosphingolipids (GSLs) [18,19]. Numerous studies indicate that GSLs play a critical role in the structural and functional organization of membranes and are involved in signal transduction and cell communication pathways [20,21,22]. Moreover, it has been reported that changes in GSL content are often associated with modifications of cellular functions on both physiological and pathological processes such as development, cancer, and neurodegeneration [23,24,25]. In particular, by using the TgCRND8 mouse model of Alzheimer’s disease, we demonstrated the abnormal cortical glycohydrolases activity at both the pre-symptomatic and symptomatic stage of the disease, which could have relevant implications for the pathophysiology of the disease [26].

Hex and Gal are two lysosomal glycohydrolases which are involved in the degradation of the ganglioside Galβ1,3GalNAcβ1,4-(NeuAcα2,3)-Galβ1,4Glc-ceramide (GM1) to the ganglioside NeuAcα2,3Galβ1,4Glc-ceramide (GM3). Hex and Gal genetic deficiency causes two severe neurodegenerative LSDs known as 3GalNAcβ1,4-(NeuAcα2,3)-Galβ1,4Glc-ceramide (GM2) and GM1 gangliosidosis, respectively [27]. Strikingly, both of these enzymes have been found to be associated with the plasma membrane [28,29,30], and their co-localization with the lipid microdomains has also been demonstrated in Jurkat T-lymphocytes [31]. The authors have also demonstrated that Hex and Gal targeting to lipid microdomains increases after T-cell stimulation, suggesting their involvement in the local reorganization of ganglioside-based signaling units. In support of this, it has recently been reported that gangliosides associated with lipid microdomains may be involved in T-cell activation and that different types of T-cells require distinct gangliosides for their activation [32].

As increasing evidence demonstrates the beneficial effect of TFEB activation on promoting autophagy in neurodegenerative diseases [33], researchers have been prompted to identify molecules able to specifically activate TFEB as promising therapeutic tools. Among these, the natural compound curcumin and several monocarbonyl curcumin analogues have been shown to enhance autophagy, promoting TFEB nuclear translocation [34,35]. In particular, the curcumin derivative C1 was found to be a potent activator of TFEB without inhibiting mTOR activity [35].

In order to establish a direct link between TFEB nuclear translocation and the recruitment of glycohydrolases Hex and Gal at the cell surface level, in this study, we induced TFEB activation by using the curcumin analogue C1 on Jurkat T-cells and evaluated Hex and Gal association at the plasma membrane microdomain level. Moreover, because modifications of GSL content and plasma membrane-associated glycohydrolase activity are often associated with neurodegenerative disorders [25,26], the effect of C1 treatment on both TFEB activation and transport of glycohydrolases to the plasma membrane was also investigated on the neuroblastoma cell line SH-SY5Y.

## 2. Results

### 2.1. Jurkat Cell Stimulation Promotes TFEB Nuclear Translocation and Induces Cell Exocytosis

**Figure 1:**
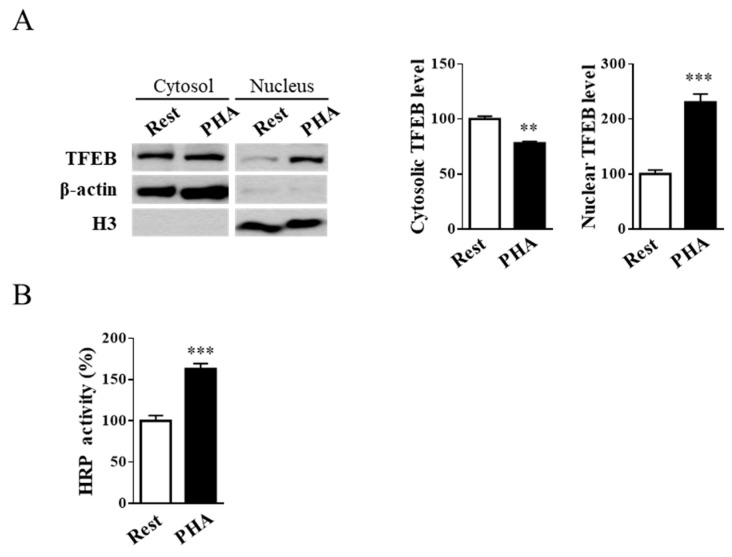
Phytohaemagglutinin (PHA)-stimulation of Jurkat cells induces transcription factor EB (TFEB) nuclear translocation and exocytosis. (**A**) Immunoblot analysis of TFEB in cytosolic and nuclear fractions from resting (Rest) and PHA-stimulated (PHA) Jurkat cells. Cytosolic TFEB level was normalized over β-actin, whereas nuclear TFEB level was normalized over H3. Values are the mean ± SEM of three independent experiments. ** *p* < 0.01 and *** *p* < 0.001 (PHA-stimulated vs. resting cells). (**B**) Horseradish peroxidase (HRP) enzyme activity in culture medium from resting and PHA-stimulated cells. Values are the mean ± SEM of three independent experiments. *** *p* < 0.001 (PHA-stimulated vs. resting cells).

As previously reported, Jurkat cell stimulation up-regulates both the expression and activity of lysosomal Hex and Gal and increases their targeting to the plasma membrane [31]. Moreover, the translocation of lysosomal glycohydrolases to the cell surface is accompanied by an increase in exocytosis [31,36]. In a recent report, Medina and collaborators [37] demonstrated that lysosomal exocytosis is regulated by TFEB. Interestingly, it has been shown that TFEB nuclear translocation induces Hex and Gal recruitment to the plasma membrane in HEK-293 cells [17]. It is noteworthy that *HEXB*, *HEXA*, and *GLB1* genes contain the CLEAR element in their promoter, making these genes putative targets of TFEB [1]. In order to investigate if T-cell activation promotes TFEB nuclear translocation, resting and phytohaemagglutinin (PHA)-stimulated Jurkat cells were treated as indicated in ‘Materials and Methods’, and cytosolic and nuclear fractions were blotted with TFEB antibody. β-Actin and H3 were used as cytosolic and nuclear markers, respectively. As reported in Figure 1A, the densitometric analysis of immunoblotting shows an increase in TFEB nuclear expression levels in PHA-stimulated with respect to resting Jurkat T-cells (*p* < 0.001), indicating the translocation of TFEB to the nucleus upon cell activation.

To verify if TFEB activation, induced by T-cell stimulation, was able to promote lysosomal exocytosis, the activity of secreted horseradish peroxidase (HRP) on the culture medium after cell stimulation was evaluated. Jurkat cells were treated with HRP and then stimulated using PHA. The results reported in Figure 1B show an increase in secreted HRP activity of approximately 1.6-fold in PHA-stimulated compared to resting cells (*p* < 0.001).

### 2.2. External Leaflet Microdomain-Associated Hex and Gal Increase after Jurkat Cell Stimulation

A great deal of evidence indicates that gangliosides associated with lipid microdomains are involved in T-cell activation and they segregate in distinct T-cell subsets following cell stimulation, resulting in asymmetric specific redistribution [25]. As previously reported [31], Jurkat T-lymphocyte stimulation up-regulates the expression and activity of both Hex and Gal and increases their targeting to lipid microdomains where they may participate in the local reorganization of GSL.

**Figure 2:**
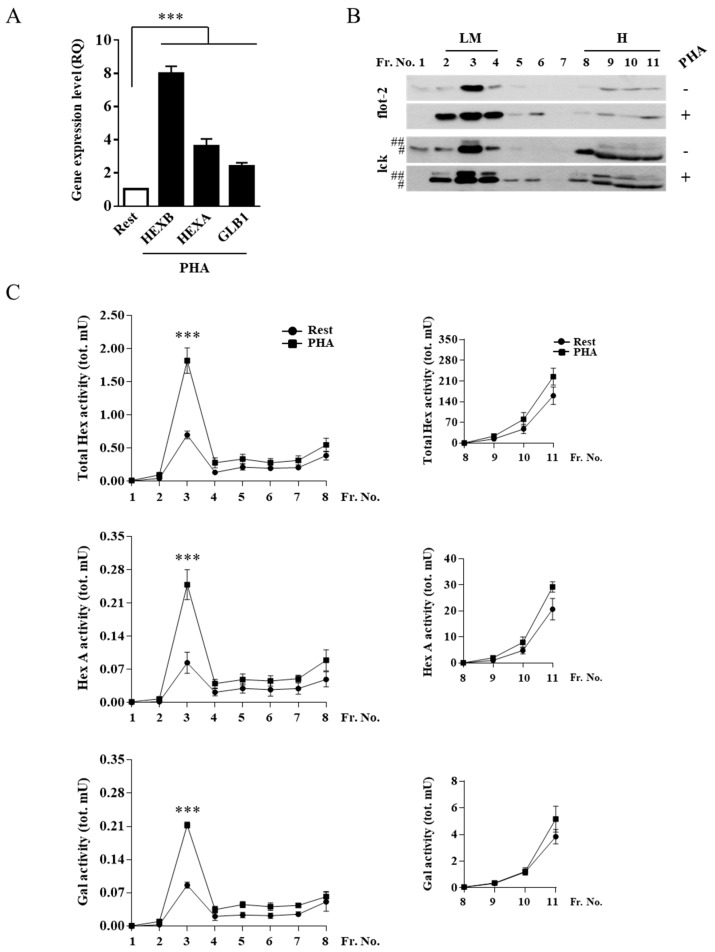
Hex and Gal glycohydrolases increase their targeting to lipid microdomains after cell stimulation. (**A**) Gene expression analysis by Q-PCR of *HEXB*, *HEXA*, and *GLB1* genes in resting and PHA-stimulated Jurkat cells. The *ACTB* gene was used as the endogenous control. The values are expressed as Relative Quantity (RQ). The mean ± SEM of three independent experiments is reported. *** *p* < 0.001 (PHA-stimulated vs. resting cells). Lipid microdomains were isolated from resting and PHA-stimulated Jurkat cells (1 × 10^8^) by a discontinuous sucrose-density gradient. (**B**) Fractions were collected from the top to the bottom of the tube and were analyzed by immunoblotting for flot-2 and lck (#, p56lck; ##, p60lck). Representative Western blotting of five independent experiments is reported. (**C**) Distribution of Total Hex, Hex A, and Gal enzymatic activities is expressed as total mU (tot. mU) in each fraction. Values are the mean ± SEM of five independent experiments. *** *p* < 0.001 (PHA-stimulated vs. resting cells). LM, lipid microdomain fractions; H, high-density fractions; Rest, resting cells; PHA, PHA-stimulated cells.

Quantitative PCR showed that there was an increase of *HEXB, HEXA*, and *GLB1* mRNA levels in stimulated Jurkat cells compared to resting cells (Figure 2A).

Moreover, total Hex, Hex A, and Gal activity in crude extract from stimulated cells was 1.5, 1.4, and 1.6-fold higher compared to resting cells, respectively, according to our previous publication [31]. To determine if the increase in Hex and Gal activity also concerns the plasma membrane-associated forms, lipid microdomains from stimulated and resting cells were isolated using a discontinuous sucrose-density gradient. Fractions collected from the top to the bottom of the tube were tested by immunoblotting analysis for the presence of the microdomain markers flotillin-2 (flot-2) and the lymphocyte-specific protein tyrosine kinase (lck). As shown in Figure 2B, flot-2 and lck were highly enriched in the light-density fractions 2–4.

The collected fractions were also assayed for the activity of Hex, both Total Hex and the Hex A isoform, using the 4-methylumbelliferyl-*N*-acetyl-β-D-glucosaminide (MUG) and the 4-methylumbelliferyl-*N*-acetyl-β-d-glucosaminide-6-sulphate (MUGS) substrates, respectively, and Gal using the 4-methylumbelliferyl-b-d-galactopyranoside (MUGal) substrate. As reported in Figure 2C, Total Hex, Hex A and Gal showed a peak of enzymatic activity corresponding to fraction 3, which co-distributed with the lipid microdomain markers. Furthermore, the increase of Total Hex, Hex A, and Gal activity in light-density fraction 3 of stimulated Jurkat cells was 2.6, 3.0, and 2,6-fold higher compared to resting cells, respectively.

**Figure 3:**
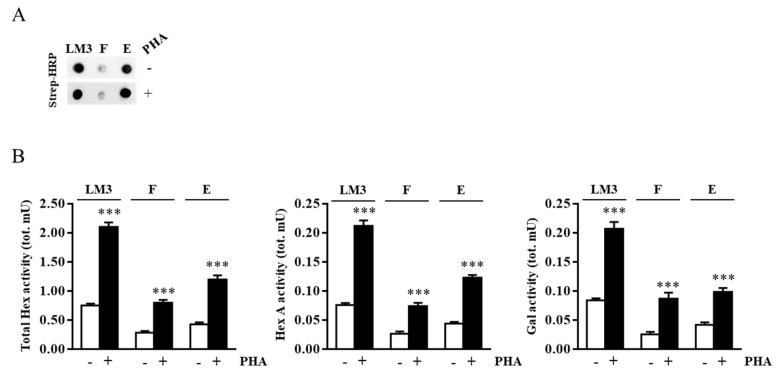
Hex and Gal are localized on external leaflet microdomains of the plasma membrane. Jurkat cells were treated with EZ-Link Sulfo-NHS-LC-Biotin to label cell surface proteins. After lipid microdomain purification, the biotinylated proteins contained in light-density fraction 3 were purified by avidin affinity chromatography. (**A**) Aliquots of concentrated and solubilized fraction 3 lipid microdomains (LM3), flow-through (F), and eluate (E) were analyzed by Dot blotting using HRP-conjugated streptavidin (Strep-HRP). (**B**) Total Hex, Hex A, and Gal enzymatic activities found in LM3, F, and E for resting (-) and PHA-stimulated (+) cells were expressed as total mU (tot. mU). The mean ± SEM of three independent experiments is reported. *** *p* < 0.001 (PHA-stimulated vs. resting cells).

As gangliosides are inserted into the external leaflet of membranes, we investigated the Hex and Gal localization in the outer leaflet of plasma membrane-lipid microdomains. For this purpose, cell surface biotinylation of Jurkat cells followed by lipid microdomains isolation was carried out. Successively, lipid microdomain proteins were recovered from flot-2-positive fraction 3, and biotinylated proteins were recovered by avidin affinity chromatography in the eluate (fraction E), as shown in Figure 3A.

The enzymatic assay of fraction E highlighted the presence of either Hex and Gal in both resting and stimulated cells, revealing their presence in the outer leaflet of plasma membrane lipid microdomains. The increase of Hex and Gal activities in stimulated Jurkat cells was also demonstrated as shown in Figure 3B. Furthermore, the presence of both Hex and Gal activity in the flow-through fraction (F) indicated that a portion of the lipid microdomain-associated enzymes was not confined on the cell surface but could be associated with the transit vesicles inside the cell.

### 2.3. TFEB Nuclear Translocation Induced by the Curcumin Analogue C1 Increases both the Expression of HEXB, HEXA, and GLB1 genes and the Recruitment of Glycohydrolases to Lipid Microdomains

In order to investigate if the observed increase of lysosomal glycohydrolases activity was correlated to the TFEB nuclear translocation rather than to an indirect effect of the Jurkat cell stimulation, we treated Jurkat cells with the curcumin analogue C1, a potent TFEB activator.

**Figure 4:**
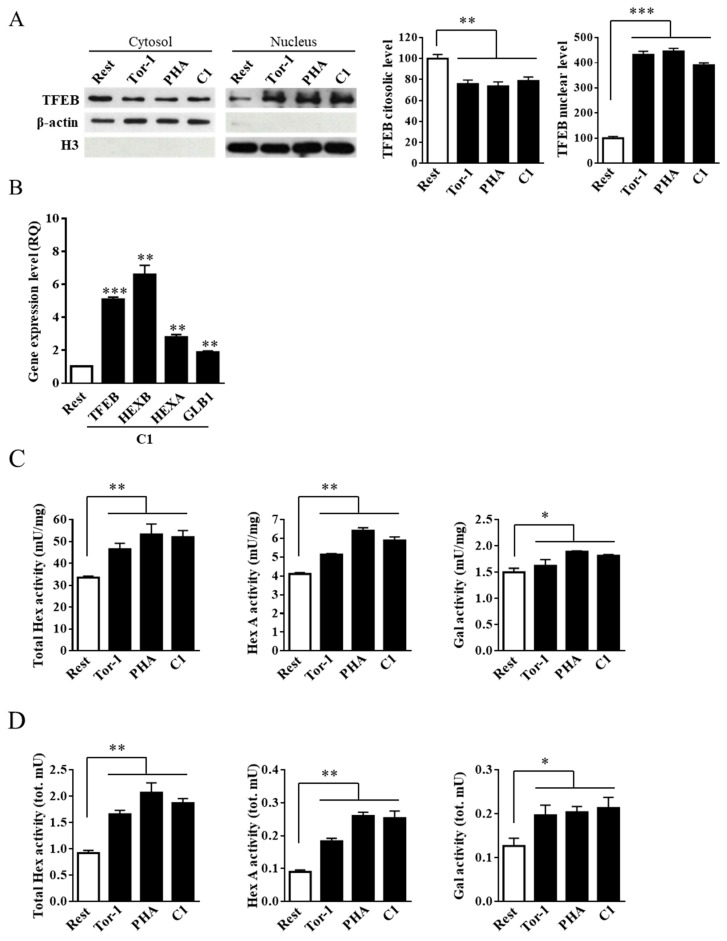
Curcumin analogue C1 promotes the recruitment of Hex and Gal on lipid microdomains by TFEB nuclear translocation in Jurkat cells. Jurkat cells were treated with PHA (24 h; 1 mg/mL), the curcumin analogue C1 (C1; 24 h, 1 μM), and torin 1 (Tor-1; 2 h, 0,1 μM,). Resting cells (Rest) were used as the negative control; torin 1 treated cells were used as the TFEB activation control. (**A**) Immunoblot analysis of cytosolic and nuclear protein fractions. TFEB level was normalized over β-actin and H3 in cytosolic and nuclear fractions, respectively. Values are the mean ± SEM of three independent experiments. *** *p* < 0.001 (treated vs. resting cells). (**B**) Gene expression analysis by Q-PCR of *TFEB*, *HEXB*, *HEXA*, and *GLB1* genes on curcumin analogue C1 (C1, 1 µM)-treated Jurkat cells. The *ACTB* gene was used as the endogenous control. Values are expressed as Relative Quantity (RQ). The mean ± SEM of three independent experiments is reported. ** *p* < 0.01 and *** *p* < 0.001 (C1 vs. resting cells). (**C**,**D**) Hex and Gal activities were assayed by using fluorogenic substrates in cell extract (mU/mg) and in the flot-2-enrhiched fractions 2–4 (tot mU), respectively. Values are the mean ± SEM of five independent experiments. * *p* < 0.1 and ** *p* < 0.01 (treated vs. resting cells).

To this end, Jurkat cells were treated for 6 h with C1 at a concentration of 1 μM, according to [35]. The cytotoxicity of C1 in Jurkat cells was assessed by (3-(4,5-dimethylthiazol-2-yl)-2,5-diphenyltetrazolium bromide) tetrazolium reduction (MTT) assay, and it was found that the compound was not toxic at the concentration used. As a positive control for TFEB nuclear translocation, cells were treated for 2 h with torin 1 at a concentration of 0.1 μM. As shown in Figure 4A, torin 1, PHA, and the curcumin analogue C1 induced TFEB nuclear translocation to a similar extent.

TFEB activation was confirmed by the increased gene expression of *HEXB*, *HEXA*, and *GLB1* on C1-treated cells. Moreover, the expression of *TFEB* gene also increased after C1 treatment (Figure 4B). As expected from the increase in *HEXB*, *HEXA*, and *GLB1* mRNAs, the C1 treatment resulted in both an increase in Hex and Gal activity (Figure 4C) and their recruitment to lipid microdomains as attested by the increase of their activities in flot-2 enriched fractions 2–4 (Figure 4D). Therefore, it appears that Jurkat cell stimulation induces TFEB nuclear translocation, which in turn promotes an increase in Hex and Gal activity and their recruitment to the cell surface.

### 2.4. Curcumin Analogue C1 Promotes TFEB Activation without Inhibiting mTORC1 Activity

**Figure 5:**
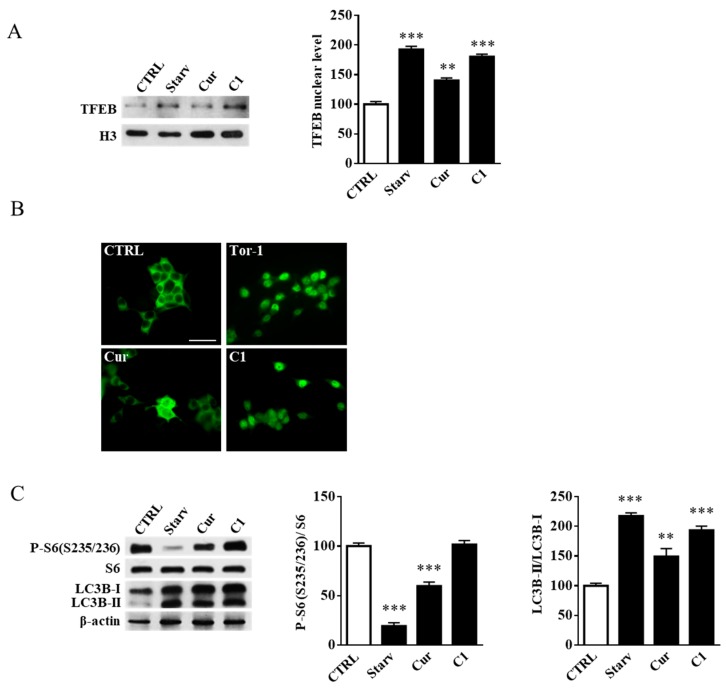
Curcumin analogue C1 promotes mechanistic target of rapamycin complex 1 (mTORC1)-independent TFEB nuclear translocation. SH-SY5Y cells were treated for 24 h with either curcumin (Cur, 5 µM) or the curcumin analogue C1 (C1, 1 µM). (**A**) Immunoblot analysis of the nuclear fractions from SH-SY5Y cells. TFEB level was normalized over H3. Starved cells were used as the positive control. Values are the mean ± SEM of three independent experiments. ** *p* < 0.01 and *** *p* < 0.001 (treated or starved vs. untreated cells, CTRL). (**B**) Immunofluorescence analysis of TFEB subcellular distribution on untreated (CTRL) and SH-SH5Y cells treated with torin 1 (Tor1), curcumin (Cur), or the curcumin analogue C1 (C1). Torin 1 was used as the positive control. Magnification, 40×; scale bar: 50 µm. (**C**) Immunoblot analysis of SH-SY5Y cell extracts. P-S6 (S235/236) was normalized over total S6, and LC3B-II was normalized over LC3B-I. β-Actin was used as the immunoblotting loading control. Values are the mean ± SEM of three independent experiments. ** *p* < 0.01 and *** *p* < 0.001 (treated or starved vs. untreated cells, CTRL).

Since TFEB activation and the associated recruitment of glycohydrolases to the plasma membrane may be relevant for the treatment of pathological conditions such as neurodegenerative disorders [26,33], the effect of C1 on TFEB activity was also investigated on a neuronal cell model. SH-SY5Y cells were treated for 24 h with curcumin or its analogue C1 at concentrations of 5 and 1 μM, respectively. Curcumin and C1 cytotoxicity in SH-SY5Y cells was assessed by MTT assay (data not shown). TFEB nuclear translocation was determined by both immunoblotting and immunofluorescence analysis. As positive controls, cells were starved for 16 h in Hank’s Balanced Salt Solution (HBSS) medium for immunoblotting analysis or treated for 2 h with torin 1 for immunofluorescence experiments. As reported in Figure 5A,B, TFEB nuclear translocation was activated by both C1 (*p* < 0.001) and, to a lesser degree, by curcumin (*p* < 0.01).

To demonstrate that C1 treatment of SH-SY5Y cells promotes TFEB nuclear translocation without effects on mTORC1 activity, we performed an immunoblotting analysis on phospho-S6 ribosomal protein (S235/236), which is known to be a p70S6K target. As reported in Figure 5C, 16 h of cell starvation strongly inhibited mTORC1 activity, as demonstrated by the reduced levels of phospho-S6 ribosomal protein. Curcumin treatment (24 h, 5 μM) showed a mild effect on the reduction of mTORC1 activity. However, even though it was possible to see a slight decrease in phospho-S6 ribosomal protein, there were no statistical differences between C1-treated (24 h, 1 μM) cells with respect to the control cells. Moreover, C1 treatment activated the autophagy flux in a manner similar to starvation as demonstrated by immunoblotting of microtubule-associated proteins 1A/1B light chain 3B (LC3B), which showed a significant increase in the autophagosome membrane-bound form LC3B-II (Figure 5C).

### 2.5. Curcumin Analogue C1 Promotes an Increase in both Expression and Activity of Hex and Gal and Their Recruitment on the Cell Surface of SH-SY5Y Cells

**Figure 6:**
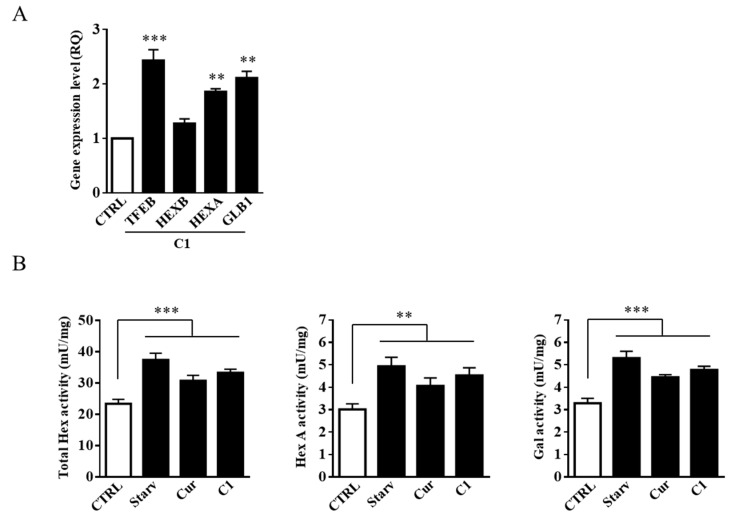
Curcumin analogue C1 promotes the expression of Hex and Gal. (**A**) Gene expression analysis by Q-PCR of *TFEB*, *HEXB*, *HEXA*, and *GLB1* genes on curcumin analogue C1 (C1, 1 µM) treated SH-SY5Y cells. The *ACTB* gene was used as the endogenous control. Values are expressed as Relative Quantity (RQ). The mean ± SEM of three independent experiments is reported. *** *p* < 0.001 (treated vs. untreated cells, CTRL). (**B**) SH-SY5Y cells were starved or treated for 24 h with either curcumin (Cur, 5 µM) or the curcumin analogue C1 (C1, 1 µM). Hex and Gal specific activities (mU/mg) were assayed by using fluorogenic substrates. Values are the mean ± SEM of five independent experiments. ** *p* < 0.01 and *** *p* < 0.001 (treated or starved vs. untreated cells, CTRL).

To demonstrate that TFEB activation is correlated with the increased expression of Hex and Gal glycohydrolases, we performed quantitative analysis on mRNA and evaluated the enzymatic activity of Hex and Gal in SH-SY5Y cells treated with 1 μM of the curcumin analogue C1 for 24 h. As reported in Figure 6A, the increased levels of *TFEB*, *HEXB*, *HEXA*. and *GLB1* mRNAs were correlated with the observed TFEB nuclear translocation promoted by C1 treatment. Moreover, as expected, the enzymatic activity of Hex, both Total Hex and Hex A, and Gal was increased after both curcumin and C1 treatments (Figure 6B).

**Figure 7:**
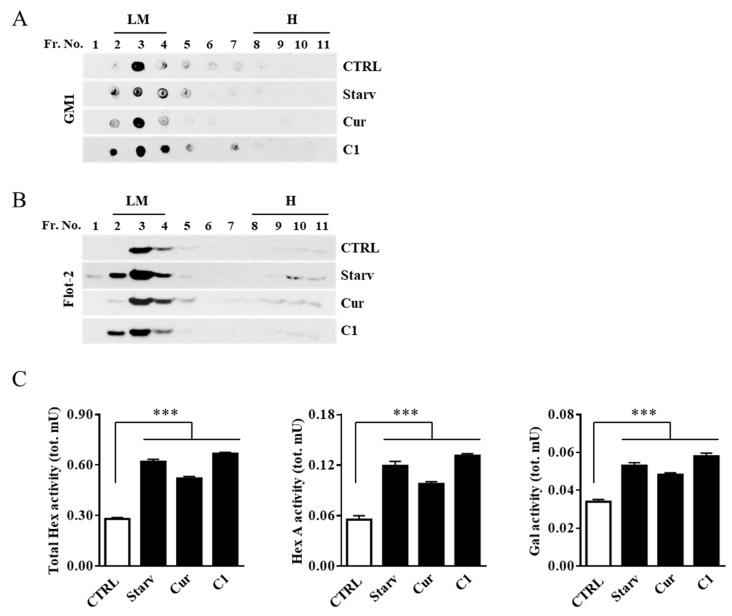
Curcumin analogue C1 promotes the recruitment of Hex and Gal on lipid microdomains. Lipid microdomains were isolated from starved (Starv), curcumin (Cur, 5 μM). and curcumin analogue C1 (C1, 1 μM) 24 h-treated and untreated (CTRL) SH-SY5Y cells. (**A**) Collected fractions were analyzed by Dot blotting for the lipid microdomain maker GM1 by using the cholera toxin B subunit. Representative Dot blotting of five independent experiments is reported. (**B**) Collected fractions were also analyzed by immunoblotting for the lipid microdomain maker flotillin 2 (Flot-2). Representative immunoblotting of five independent experiments is reported. (**C**) Total Hex, Hex A, and Gal enzymatic activities in the GM1-enrhiched fractions 2–4 are reported as total mU (tot. mU). Values are the mean ± SEM of five independent experiments. *** *p* < 0.001 (treated or starved vs. untreated cells, CTRL). LM, lipid microdomain fractions; H, high-density fractions.

To analyze the effect of the curcumin and C1 treatment on plasma membrane-associated glycohydrolase levels, we purified and recovered lipid microdomains. Fractions from the gradient were analyzed for the presence of the specific microdomain markers GM1, by dot blotting, and flot-2, by immunoblotting analysis. As shown in Figure 7A,B, GM1 and flot-2 were enriched in the light-density fractions 2–4 which were then collected and assayed for Hex, both Total Hex and Hex A, and Gal activities. As reported in Figure 7C, the activity of Hex and Gal was strongly increased in flot-2-positive fractions 2–4 of starved and C1-treated cells and, to a lesser extent, in curcumin-treated cells.

**Figure 8:**
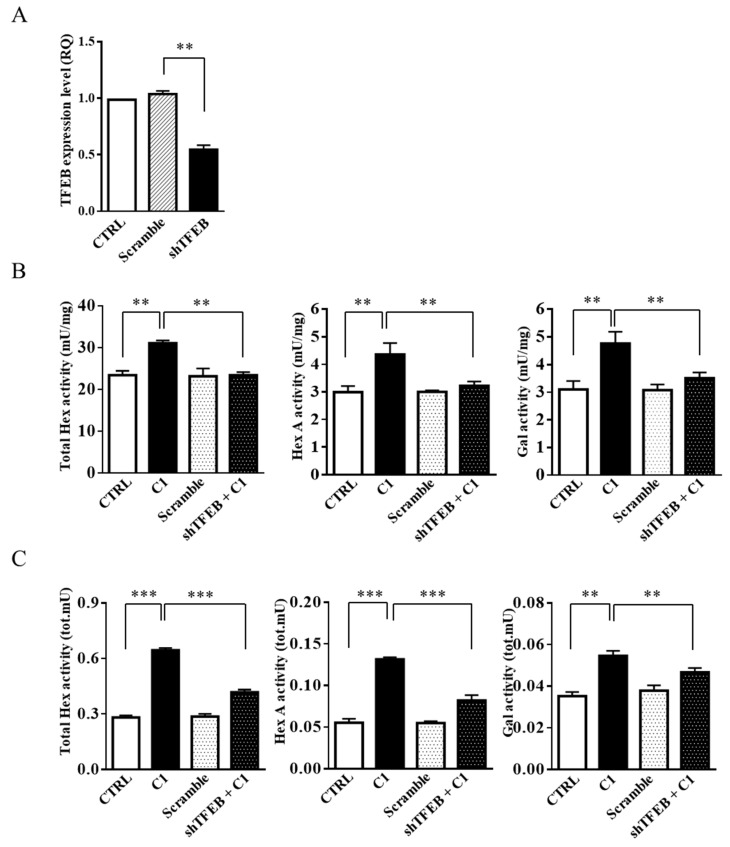
Curcumin analogue C1 failed to increase Hex and Gal activity and their recruitment on lipid microdomains in TFEB knock-down cells. SH-SY5Y cells were transfected with shRNA for TFEB (shTFEB) or scrambled shRNA (Scramble) as the control. (**A**) Gene expression analysis by Q-PCR of the *TFEB* gene in SH-SY5Y (CTRL), Scramble, and shTFEB cells. The values are expressed as Relative Quantity (RQ). The mean ± SEM of three independent experiments is reported. ** *p* < 0.01 (Scramble vs. shTFEB). (**B**,**C**) SH-SY5Y (C1) and shTFEB (shTFEB + C1) cells were treated for 24 h with the curcumin analogue C1 (C1, 1 µM); untreated SH-SY5Y (CTRL) and untreated scramble (Scramble) cells are reported as controls. Hex and Gal activities were assayed by using fluorogenic substrates in cell extract (mU/mg) and in the flot-2-enrhiched fractions 2–4 (tot mU), respectively. Values are the mean ± SEM of five independent experiments. ** *p* < 0.01 and *** *p* < 0.001 (treated vs. untreated SH-SY5Y cells or treated shTFEB vs. treated SH-SY5Y cells).

Finally, in order to unambiguously demonstrate that the effect of the curcumin analogue C1 on the recruitment of lysosomal glycohydrolases to the plasma membrane was associated with TFEB activation, SH-SY5Y cells were transfected with shRNA for TFEB. As shown in Figure 8B, C1 treatment of TFEB knock-down cells failed to promote the increase of Hex and Gal enzymatic activity. Moreover, after C1 cell treatment, the recruitment of the glycohydrolases to the cell surface, even if slightly increased with respect to the scramble cells, was significantly lower than in normal cells (Figure 8C). These results strongly support the hypothesis of a correlation between TFEB activation and the recruitment of lysosomal glycohydrolases to the plasma membrane.

## 3. Discussion

Curcumin is a natural polyphenol, derived from the turmeric *Curcuma longa*, showing several pharmacological activities. Recently, it has been demonstrated that curcumin can induce autophagy through inhibition of the Akt-mTOR pathway [38] and by directly binding to TFEB, promoting its nuclear translocation [34].

In this study, by using the human neuroblastoma cell line SH-SY5Y, which is a widely used cell model in the study of autophagic pathways and neurodegeneration [39,40,41], we confirmed that curcumin has an inhibitory effect on mTORC1 and promotes both lysosomal functions and autophagy by inducing TFEB nuclear translocation. Moreover, the curcumin-dependent TFEB activation was accompanied by an increase in both lysosomal enzyme Hex and Gal activity and their recruitment to the plasma membrane, where these enzymes may be involved in the in situ remodeling of GSL [42]. Notably, we previously demonstrated the alteration of lysosome-to-plasma membrane transport in the TgCRND8 mouse model of Alzheimer’s disease, revealing an abnormal localization of glycohydrolases in post-synaptic density microdomains starting from the pre-symptomatic stage of the disease [26]. Interestingly, a role played by ganglioside metabolism on the pathogenesis of the disease has been reported [25]. Moreover, the involvement of TFEB in most neurodegenerative diseases has been extensively documented [11,12,15,16].

Moreover, in this study we demonstrated for the first time that PHA stimulation of Jurkat T-lymphocytes induced TFEB nuclear translocation and resulted in both an increase of the enzymatic activity of Hex and Gal and their targeting to the plasma membrane as promoted by lysosomal exocytosis. The treatment of Jurkat cells with the curcumin analogue C1, a potent mTORC1-independent TFEB activator [35], also induced TFEB nuclear translocation, increased Hex and Gal expression and activity, and enhanced plasma membrane-associated glycohydrolases in an extent very similar to the PHA-stimulated cells. These results clearly indicate that the TFEB nuclear translocation induced by Jurkat cell stimulation is closely associated with the delivery of lysosomal glycohydrolases to the cell surface and support the hypothesis of a possible involvement of TFEB in T-lymphocyte stimulation. Of note is that the implication of TFEB in the immune response has recently been reviewed [43], and the involvement of specific gangliosides in T-cell activation has also been reported [32]. Furthermore, our results suggest that lysosomal exocytosis may be relevant not only for cellular clearance and membrane repair but also for not yet clearly elucidated processes which require in situ remodeling of GSL.

The treatment of the neuroblastoma cell line SH-SY5Y with the curcumin analogue C1 also promoted TFEB nuclear translocation and the increase of lysosomal glycohydrolase expression and activity and their recruitment to the cell surface. Additionally, by a *TFEB* silencing experiment, we clearly demonstrated the ability of C1 to induce the transport of Hex and Gal to the plasma membrane by direct TFEB activation. In brief, the overall results (i) provide strong evidence of the correlation between TFEB nuclear translocation and the recruitment of Hex and Gal glycohydrolases to the plasma membrane; and (ii) demonstrate the ability of C1 to promote the recruitment of lysosomal glycohydrolases to the plasma membrane microdomains via an mTORC1-indipendent TFEB activation mechanism.

In conclusion, because C1 directly targets TFEB, we herein unambiguously demonstrated the link between TFEB activation, which promotes lysosome-to-plasma membrane fusion, and the transport of active forms of lysosomal glycohydrolases to the cell surface. The role of plasma membrane glycohydrolases has not yet been fully elucidated, but their implication in remodeling the glycosphingolipid pattern has been clearly confirmed [18,19]. Based on the role played by TFEB in recruiting lysosomal glycohydrolases to the plasma membrane, this transcription factor may represent an effective target to modulate this pathway.

Moreover, by using the SH-SY5Y neuroblastoma cell line, we confirmed the ability of the curcumin analogue C1 to activate TFEB without inhibiting mTORC1 activity, thus suggesting its potential therapeutic efficacy for the treatment of neurodegenerative diseases by promoting autophagy.

## 4. Materials and Methods

### 4.1. C1 Synthesis

C1 was synthesized by a condensation reaction between anhydrous acetone (17.2 mmol) and o-methoxybenzaldehyde (36.1 mmol) in anhydrous methanol (30 mL), using sodium methoxide (37.8 nmol) as a deprotonating base, following the synthetic procedure previously reported in literature [44]. Purification was performed by column chromatography over silica gel (Cyclohexane/CH_3_OH) and subsequent crystallization by methanol. Yield: 89%; m.p.: 119–121 °C; purity ≥ 98%; single spot TLC and NMR analysis (Figures S1 and S2).

### 4.2. Cell Culturing

Jurkat T-lymphocytes (JE6-1) and the SH-SY5Y cell line (ATCC, Manassas, VA, USA) were cultured in RPMI 1640 (Gibco) and DMEM (Gibco) media, respectively, both supplemented with 10% (*v*/*v*) heat-inactivated bovine fetal serum (FBS), 2 mM l-glutamine, 100 units/mL penicillin, and 100 mg/mL streptomycin. Starvation was performed in HBSS medium (Sigma-Aldrich, St. Louis, MO, USA), with Ca^2+^ and Mg^2+^, supplemented with 10 mM HEPES.

### 4.3. Drugs and Cell Treatments

The cells were treated for 24 h with the following drugs: PHA (1 mg/mL) from Sigma-Aldrich; curcumin (5 µM) from Sigma-Aldrich; and curcumin analogue C1 (1 µM). Cells were treated for 2 h with torin 1 (0.1 µM) from Cell Signaling.

### 4.4. TFEB RNA Interference

TFEB RNAi was performed by using shRNA expression constructs which were purchased from Origene (Rockville, MD, USA). A scrambled shRNA was used as the control. Briefly, SH-SY5Y cells were transfected using Lipofectamine LTX (Invitrogen). Stable transfected cells were obtained using 0.1 mg/mL puromycin (Sigma).

### 4.5. Cytosolic, Nuclear, and Enriched Plasma Membrane Extracts

Cytosolic and nuclear fractions were isolated from resting and PHA-stimulated Jurkat cells [17] and from SH-SY5Y cells treated for 24 h with curcumin and C1, or after starvation, as previously described. Plasma membrane proteins were isolated from soluble proteins using the Mem-PER Eukaryotic Membrane Protein Extraction Kit (Pierce, Thermo Scientific, Waltham, MA, USA) in accordance with the manufacturer’s procedure. Protein concentration was determined by Bradford’s assay.

### 4.6. Isolation of Lipid Microdomains

Lipid microdomains from Jurkat and SH-SY5Y cells were isolated by discontinuous sucrose-density gradient centrifugation as previously reported [31]. After centrifugation, eleven fractions of 450 μL were collected from the top to the bottom of the tubes.

### 4.7. Quantitative PCR

Total RNA was extracted from 2 × 10^6^ Jurkat or SH-SY5Y cells using a standard Trizol protocol (Sigma-Aldrich). Isolated RNA was treated with a TURBO DNA-free™ Kit (ThermoFisher) according to the manufacturer’s procedure. cDNA was obtained by reverse transcription of 2 µg of RNA using a Maxima H Minus First Strand cDNA Synthesis Kit (Thermo Fisher) according to the manufacturer’s procedure. cDNA was used for the evaluation of *HEXB*, *HEXA*, *GLB1*, and *TFEB* gene expression by quantitative PCR (Q-PCR) in a Stratagene Mx3000P Q-PCR machine (Agilent Technologies) as previously reported [31]. Data were analyzed by the ΔΔ*C*t method. The sequences of specific primers used in this work are listed in Table 1.

### 4.8. Horseradish Peroxidase Assay

Jurkat cells (1 × 10^6^/mL) were treated for 2 h with 2 mg/mL of HRP (Sigma-Aldrich). Successively, cells were recovered, washed with Dulbecco’s PBS (DPBS), and resuspended in complete media. Cells were incubated for 16 h and then stimulated or not (resting) with PHA for 24 h. The culture medium was recovered, and HRP activity was determined as previously reported [45]. One unit (U) corresponds to the amount of enzyme that oxidizes 1 µmol of substrate/min at pH 5.0 at 25 °C.

### 4.9. Isolation of Cell Surface Lipid Microdomain Proteins

Cell surface proteins of resting and PHA-stimulated Jurkat cells (1 × 10^8^) were biotinylated using 1 mg/mL of EZ-Link Sulfo-NHS-LC-Biotin (Thermo Scientific), and the reaction was performed according to the manufacturer’s procedure.

Cell surface lipid microdomain protein isolation was performed as previously reported [28]. Briefly, after discontinuous sucrose-density gradient centrifugation, flot-2-positive fraction 3 was recovered from the ultracentrifugation tube and diluted 1:4 with 10 mM Tris, 150 mM NaCl, 5 mM EDTA (TNE), pH 7.4, containing 1% (*v*/*v*) Triton X-100 (TX-100). Lipid microdomain vesicles were recovered by ultracentrifugation at 60,000 rpm at 4 °C for 2 h using a TLA-100.3 rotor and an Optima Max ultracentrifuge. The pellet was resuspended in 100 µl of PBS containing 1% (*v*/*v*) TX-100 and disaggregated by incubating for 10 min at 37 °C (LM3). Lipid microdomain proteins were loaded at the top of a 0.5 mL column containing the UltraLink™ Monomeric Avidin resin (Thermo Scientific). After washing, biotinylated proteins were eluted by 5 mM d-biotin in PBS.

### 4.10. Immunoblotting and Dot Blot Analysis

The protein extracts from cytosolic, nuclear, and sucrose-density gradient fractions were subjected to SDS-PAGE [46]. Separated proteins were transferred to the PVDF membrane (Biorad, Hercules, CA, USA), blocked in 50 mM Tris-HCl, 150 mM NaCl (TBS), pH 7.6, containing 5% (*w*/*v*) BSA/0.1% (*v*/*v*) Tween 20 and reacted over-night at 4 °C with one of the primary antibodies reported in Table 2. After being washed, blots were incubated with the appropriate HRP-conjugated secondary antibody and developed by an ECL detection system (GE Healthcare, Chicago, IL, USA). Western blot images were acquired using an ImageScanner calibrated densitometer (Amersham Pharmacia), and densitometry analysis was performed using ImageJ.

GM1 ganglioside was revealed by Dot Blot analysis as reported in [31].

### 4.11. Determination of Enzyme Activities

Total Hex, Hex A, and Gal activities were determined by using the artificial substrates MUG, MUGS, and MUGal (Sigma-Aldrich) as previously reported [31].

One enzymatic unit (U) corresponds to the amount of enzyme that hydrolyses 1 mmol of substrate/min at 37 °C.

### 4.12. Immunofluorescence Analysis

SH-SY5Y cells were plated on glass coverslips, previously coated with poly-l-lysine (Sigma-Aldrich), for 30 min at RT, and incubated for 24 h in DMEM media before the drug treatments. The cells were fixed with 4% paraformaldehyde/DPBS for 20 min at RT, washed three times with DPBS, and blocked in DPBS containing 5% (*v*/*v*) FBS and 0.3% (*v*/*v*) TX-100 for 1 h at RT. After further washings, the cells were incubated for 1 h in the antibody solution (D-PBS, 1% (*w*/*v*) BSA, 0.3% (*v*/*v*) Tx-100) with the rabbit anti-TFEB primary antibody (1:600, Bethyl Laboratories, Cat. n. A303-673A). After being washed, the cells were incubated with the donkey anti-rabbit IgG Alexa Fluor^®^488 secondary antibody (Thermo-Fisher, Cat. n. A-21206) for 1 h in an antibody solution. Successively, the coverslips were mounted on glass slides using Vectashield with DAPI (Vector Laboratories Inc. Burlingame, CA, USA), and fluorescence microscopy analysis was performed using a Nikon TE2000 microscope (Nikon Instruments S.p.A, Florence, Italy). Image processing was performed by using Adobe Photoshop CS software (Adobe Systems Incorporated).

### 4.13. Statistical Analysis

All data were expressed as mean ± SEM. Statistical differences were evaluated using unpaired Student’s t-test. The threshold for statistical significance was set at *p* < 0.05.

## Figures and Tables

**Table 1:** List of primer sequences used for Q-PCR analysis.

Target Gene (Human)		Sequence (5′→3′)
*HEXB*	Sense	TTTGGGAGGAGATGAAGTGG
	Antisense	AAACCTCCTGCCAGACAATG
*HEXA*	Sense	GCATTTGAAGGTACCCCTGA
	Antisense	TCAACTTGTTGCTCCACAGC
*GLB1*	Sense	GTTATAACAGTGCAGGTTGAAAATGAA
	Antisense	CCCAGATGGTGGCGAAAG
*TFEB*	Sense	TCTGCAGCAGTCGCAGCAT
	Antisense	CCAATGTGCAGCATGGCCA
*ACTB*	Sense	AGAAAATCTGGCACCACACC
	Antisense	GGGGTGTTGAAGGTCTCAAA

**Table 2:** List of primary antibodies used for immunoblot analysis.

Target	Type	Producer (Cat. Number)	Working Dilution
TFEB	Goat IgG	Abcam (ab2636)	1:1000
H3	Rabbit IgG	Abcam (ab1791)	1:5000
β-actin	Mouse IgG2a	Sigma-Aldrich (A5316)	1:5000
LC3B	Rabbit IgG	Cell Signaling Technology (#2775)	1:1000
flotillin-2	Mouse IgG1	BD Biosciences (610383)	1:5000
Lck	Mouse IgG2b	Santa Cruz Biotechnology (sc-433)	1:200
Phospho-S6 ribosomal protein (S235/236)	Rabbit IgG	Cell Signaling Technology (#2211)	1:1000
S6 ribosomal protein	Rabbit IgG	Cell Signaling Technology (#2217)	1:1000

## Supplementary Materials

Supplementary materials can be found at https://www.mdpi.com/1422-0067/20/6/1363/s1. Figure S1: 1H NMR spectra of C1 in deuterated chloroform CDCl; Figure S2: 13C NMR spectra of C1 in deuterated chloroform.
