# Supplementary figures and images for: Curcumin Analogue C1 Promotes Hex and Gal Recruitment to the Plasma Membrane via mTORC1-Independent TFEB Activation
Source: Int J Mol Sci. 2019 Mar 18;20(6):1363. doi: 10.3390/ijms20061363 (PMC6471159; doi:10.3390/ijms20061363)

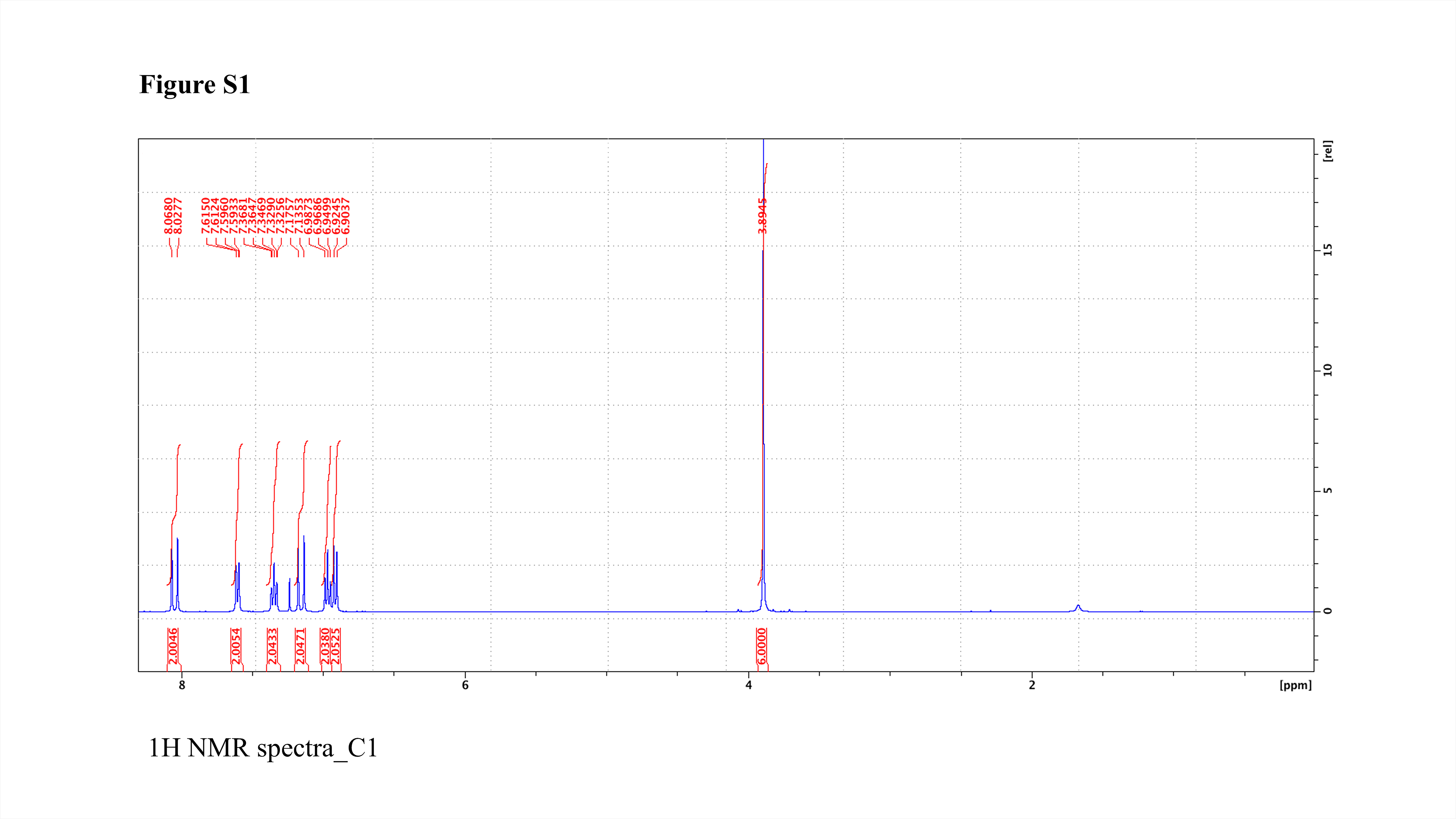

Supplement: Supplementary file 1 [file ijms-20-01363-s001.zip › Supplementary Figures/Figure S1.tif]

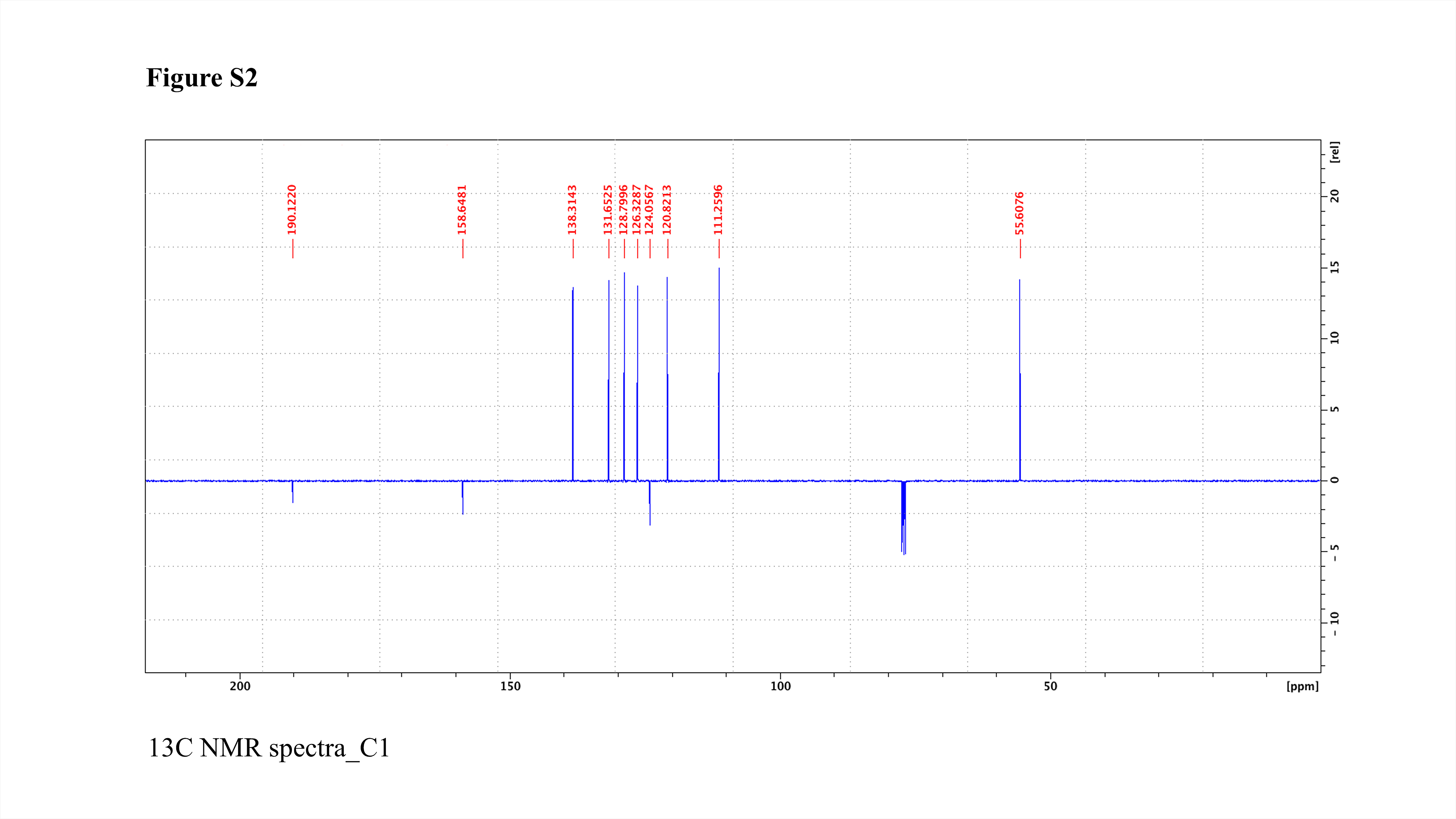

Supplement: Supplementary file 1 [file ijms-20-01363-s001.zip › Supplementary Figures/Figure S2.tif]
